# LAG3 is an independent prognostic biomarker and potential target for immune checkpoint inhibitors in malignant pleural mesothelioma: a retrospective study

**DOI:** 10.1186/s12885-023-11636-1

**Published:** 2023-12-07

**Authors:** Ken Arimura, Kenzo Hiroshima, Yoji Nagashima, Tadao Nakazawa, Akira Ogihara, Mami Orimo, Yasuto Sato, Hideki Katsura, Masato Kanzaki, Mitsuko Kondo, Etsuko Tagaya

**Affiliations:** 1 Department of Respiratory Medicine, Tokyo Women’s Medical University, Tokyo, Japan; 2 Department of Pathology, Tokyo Women’s Medical University Yachiyo Medical Center, Chiba, Japan; 3 Department of Surgical Pathology, Tokyo Women’s Medical University, Tokyo, Japan; 4 Department of Thoracic Surgery, Tokyo Women’s Medical University, Tokyo, Japan; 5 Graduate School of Public Health, Shizuoka Graduate University of Public Health, Shizuoka, Japan

**Keywords:** LAG3, Immune checkpoint inhibitors, Malignant pleural mesothelioma, Novel prognostic biomarker, TCGA, mRNA, IHC

## Abstract

**Background:**

Lymphocyte-activation gene 3 (LAG3) is an immune checkpoint receptor; novel LAG3 immune checkpoint inhibitors (ICIs) exhibit therapeutic activity in melanoma. The role of LAG3and ICIs of LAG3 are unknown in malignant pleural mesothelioma (MPM). This study aimed to uncover the prognostic landscape of LAG3 in multiple cancers and investigate the potential of using LAG3 as an ICIs target in patients with MPM.

**Methods:**

We used The Cancer Genome Atlas (TCGA) cohort for assessing mRNA expression and our cohort for immunohistochemical expression. TCGA cohort were analyzed using the Wilcoxon rank-sum test to compare mRNA expression between normal and tumor tissues in multiple cancers. We used 86 MPM cases from TCGA and 38 MPM cases from our cohort to analyze the expression of LAG3 in tumor-infiltrating lymphocytes. The mean *LAG3* mRNA expression was set as the cut-off and samples were classified as positive/negative for immunohistochemical expression. Overall survival (OS) of patients with MPM was determined using the Kaplan–Meier method based on LAG3 mRNA and immunohistochemical expression. OS analysis was performed using the multivariate Cox proportional hazards model. The correlation of *LAG3* expression and mRNA expression of tumor immune infiltration cells (TIICs) gene markers were estimated using Spearman correlation. To identify factors affecting the correlation of *LAG3* mRNA expression, a multivariate linear regression model was performed.

**Results:**

*LAG3* mRNA was associated with prognosis in multiple cancers. Elevated *LAG3* mRNA expression was correlated with a better prognosis in MPM. LAG3 expression was detected immunohistochemically in the membrane of infiltrating lymphocytes in MPM. LAG3 immunohistochemical expression was correlated with a better prognosis in MPM. The multivariate Cox proportional hazards model revealed that elevated LAG3 immunohistochemical expression indicated a better prognosis. In addition, *LAG3* mRNA expression was correlated with the expression of various gene markers of TIICs, the most relevant to programmed cell death 1 (PD-1) with the multivariate linear regression model in MPM.

**Conclusions:**

LAG3 expression was correlated with prognosis in multiple cancers, particularly MPM; LAG3 is an independent prognostic biomarker of MPM. LAG3 regulates cancer immunity and is a potential target for ICIs therapy. PD-1 and LAG3 inhibitors may contribute to a better prognosis in MPM.

**Trial registration:**

This study was registered with UMIN000049240 (registration day: August 19, 2022) and approved by the Institutional Review Board (approval date: August 22, 2022; approval number: 2022–0048) at Tokyo Women’s Medical University.

**Supplementary Information:**

The online version contains supplementary material available at 10.1186/s12885-023-11636-1.

## Background

Malignant pleural mesothelioma (MPM) is a rare tumor [1, 2] with poor prognosis. It is associated with asbestos exposure [3, 4]; it originates from the transformation of the pleural mesothelial cells [5]. Mesothelial cells transform into to MPM after a latency period of 30–40 years [6, 7]. Although several hypotheses have been proposed, the mechanism of tumorigenesis in MPM remains unknown [3, 5]. Some potential prognostic biomarkers and molecular targets of MPM have been identified [8–10]; however, these targets are not conclusive. Furthermore, treatment options for MPM are limited. Standard therapy for MPM includes chemotherapy with cisplatin and pemetrexed [11] and combination immune checkpoint inhibitors (ICIs) with nivolumab and ipilimumab [12]. However, many patients develop resistance, and the overall survival (OS) with treatment is 12–18 months [11, 12]. In addition to research on the mechanisms of resistance and progression, identifying novel prognostic biomarkers and molecular targets is essential to improve the OS of patients with MPM. Recently, the combination therapy of two ICIs, relatlimab and nivolumab, altered progression-free survival (PFS) compared with nivolumab-alone treatment for patients with unresectable or untreated metastatic melanoma [13]. Relatlimab is a novel ICI that targets lymphocyte-activation gene 3 (LAG3) [13]. LAG3 was first reported in 1990 [14] and is an important immune checkpoint expressed on the membranes of tumor-infiltrating lymphocytes [13, 15]. LAG3 includes domains that bind major histocompatibility complex class II with high affinity and contribute to escape from the cancer immune system. LAG3inhibits the proliferation, activation, homeostasis, and functions of CD4 + and CD8 + T cells [15, 16]. High expression of LAG3 correlates with a poor prognosis in renal clear cell carcinoma, primary central nervous system lymphoma, and muscle-invasive bladder cancer. However, it correlates with a better prognosis in gastric cancer and melanoma [17]. Although the role of LAG3 expression in tumor prognosis, including MPM, is limited, some studies suggest that LAG3 expression was correlated with the prognosis of patients with MPM [18, 19]. Furthermore, various clinical trials (including phase 1 MPM trials) on LAG3 inhibitors with or without programmed cell death 1 (PD-1*/PDCD1*) or programmed cell death ligand 1 (PD-L1*/CD274*) inhibitors are ongoing [13, 20–22]. The correlation between *LAG3* expression and mRNA expression of tumor immune infiltration cells (TIICs) gene markers in MPM is indefinite. Therefore, we conducted this study to determine whether LAG3 is a potential prognostic biomarker for various cancers or a molecular therapeutic target for MPM. In addition, we explored new insights into the correlation between *LAG3* expression and the expression of gene markers of TIICs in MPM. The specific purpose of this study was to assess *LAG3* expression correlations with OS and the expression of gene markers of TIICs in MPM.

## Methods

### The Cancer Genome Atlas (TCGA) Cohort

*LAG3* mRNA expression in TCGA pan-cancer cohort, including 36 various human cancer types, was extracted to study their association with OS (https://xena.ucsc.edu/) [23]. In addition, the cohort was used to compare the mRNA expression between normal and tumor tissues of 17 types with TIMER to visualize and analyze the data from TCGA (https://cistrome.shinyapps.io/timer/) [24]. The mRNA expression was changed to log2 of transcripts per million to evaluate the difference in mRNA expression between tumor tissues and normal tissues adjacent to tumor tissues [25]. A cut-off value was set as the mean for *LAG3* expression to compare the expression (high and low) of *LAG3* mRNA in TCGA cohort. In addition, clinical characteristics, including age, sex, stage, and histology, were extracted in TCGA cohort. The correlation of *LAG3* mRNA expression with mRNA expression of gene markers of TIICs in TCGA cohort was assessed [25, 26]: immune checkpoints (*CD274*, *PDCD1*, *CTLA4*); macrophages (*CD68*), M1-type (classically activated) macrophages (*NOS2*), M2-type (alternatively activated) macrophages (*ARG1*, *MRC1*); tumor-associated macrophages (*HLA-G*, *CD80*, *CD86*); monocytes (*CD14*); natural killer cells (*XCL1*, *KIR3DL1*, *CD7*); neutrophils (*MPO*); dendritic cells (*CD1C*); B cells (*CD19*, *CD38*); CD8 + T cells (*CD8A*, *CD8B*); follicular helper T cells (*CXCR5*, *ICOS*, *BCL6*); T helper-1 cells (*IL12RB2*); T helper-2 cells (*CCR3*, *STAT6*, *GATA3*); T helper-9 cells (*TGFBR2*, *IRF4*, *SPI1*); T helper-17 cells (*IL-21R*, *IL-23R*, *STAT3*); T helper-22 cells (*CCR10*, *AHR*); regulatory T cells (*FOXP3*, *CCR8*).

### Immunohistochemistry (IHC) for MPM

We analyzed all 38 clinicopathologically diagnosed MPM cases by obtaining surgical or biopsy samples at Tokyo Women’s Medical University Hospital and Tokyo Women’s Medical University Yachiyo Medical Center from March 9, 2000, to June 12, 2020. In addition, data on clinical characteristics were collected from the patient’s medical records. Patient characteristics, including age, sex, stage, histology, smoking history, and the results of respiratory function tests, are summarized. Formalin-fixed paraffin-embedded tissues were stained with a primary antibody against LAG3 (#15,372, 1:100, rabbit monoclonal, Cell Signaling Technology, Danvers, MA, USA) by IHC using an autostainer (BOND-MAX, Leica Biosystems, Wetzlar, Germany) after sectioning the samples to 4 μm slices. The tissue slides were heated for 20 min in BOND Epitope Retrieval Solution 2 (Leica Biosystems) before staining to improve the intensity. Primary antibody binding tissue sections were visualized using BOND Polymer Refine Detection (Leica Biosystems), including anti-mouse IgG antibody as a secondary antibody and 3,3′-diaminobenzidine as a substrate. IHC staining for LAG3 was defined as positive when the proportion of morphologically confirmed positive lymphocytes was 1% or greater within MPM [11]. LAG3 expression was evaluated by a well-experienced pathologist (KH) and oncologist (KA). If the assessments were dissimilar, the two evaluators discussed amongst themselves to reach an agreement [27].

For immunofluorescence, the tissues were stained with primary antibodies against LAG3 (#209236, 1:100, rabbit monoclonal, Abcam, Cambridge, UK), CD4 (#NCL-L-CD4-368, 1:100, mouse monoclonal, Leica Biosystems, Newcastle, UK), and CD8 (#NCL-L-CD8-4B11, 1:50, mouse monoclonal, Leica Biosystems) using an autostainer (BOND-MAX) after sectioning the samples to 4 μm slices. Before staining, the tissue slides were heated for 20 min in BOND Epitope Retrieval Solution 2 (#AR9640, Leica Biosystems). The following were applied for visualization: anti-mouse IgG antibody as a secondary antibody, HRP conjugated anti-rabbit IgG antibody (#AR9640, Leica Biosystems) as a secondary antibody or a polymer, Opal 520 for CD4, Opal 570 for CD8, and Opal 690 for LAG3 (#NEL810001KT, Opal 4-Color IHC Kit, Akoya Biosciences, Marlborough, MA, USA). The nuclei were counterstained using 4′,6-diamidino-2-phenylindole (DAPI) (#340-07971, 1:5000; Dojindo Laboratories, Kumamoto, Japan).

### Statistical analyses

Data analysis was performed using R version 3.6.2 (The R Foundation for Statistical Computing, Vienna, Austria), Graph Pad PRISM 9 (GraphPad Software, La Jolla, CA, USA), or JMP17 (SAS Institute, Cary, NC, USA). Comparison of mRNA expression between normal and tumor tissues in the TCGA cohort was by using the Wilcoxon rank-sum test. The association between *LAG3* mRNA and LAG3 immunohistochemical expression and clinical variable was estimated using the Wilcoxon signed-rank test. OS related to *LAG3*, *PDCD1, and CTLA4* expression (high/low groups in mRNA expression and LAG3 positive/negative groups in the immunohistochemical expression) was estimated using the Kaplan–Meier method and assessed for significance using the log-rank test. We measured the effect of high/low *LAG3* mRNA expression or positive/negative LAG3 immunohistochemical expression and clinical variables including age, sex, stage, and histological type on death over time. Using a univariate Cox proportional hazards model, we reported the hazard ratio (HR) with a 95% confidence interval (CI). A multivariate Cox proportional hazards model (adjusted for clinical variables including age, sex, and stage as basic data elements and given a limited number of patients) was used to investigate the association between OS and high/low *LAG3* mRNA expression or LAG3 positive/negative immunohistochemical expression.

The correlation between *LAG3* mRNA expression and the mRNA expression of gene markers of TIICs was estimated using Spearman’s rank sum test with r, which is R-value was obtained [26]. The level of r was determined with the following absolute values: very weak, 0.001– ± 0.19; weak, ± 0.20–0.39; moderate, ± 0.40–0.59; strong, ± 0.60–0.79; very strong, ± 0.80–1.0. Two-sided *P* values < 0.05 indicated statistical significance. We measured the effect of the correlation between *LAG3* mRNA expression and mRNA expression on TIICs gene markers. We used a multivariate linear regression model (adjusted for 8 gene markers: 5 gene markers of high correlation with *LAG3* mRNA and 3 gene markers for immune checkpoint) to identify factors affecting the correlation of *LAG3* mRNA expression usingβ, the regression coefficient, along with the 95% CI.

## Results

### *LAG3* mRNA Expression in Various Human Cancers in TCGA Cohort

**Fig. 1:**
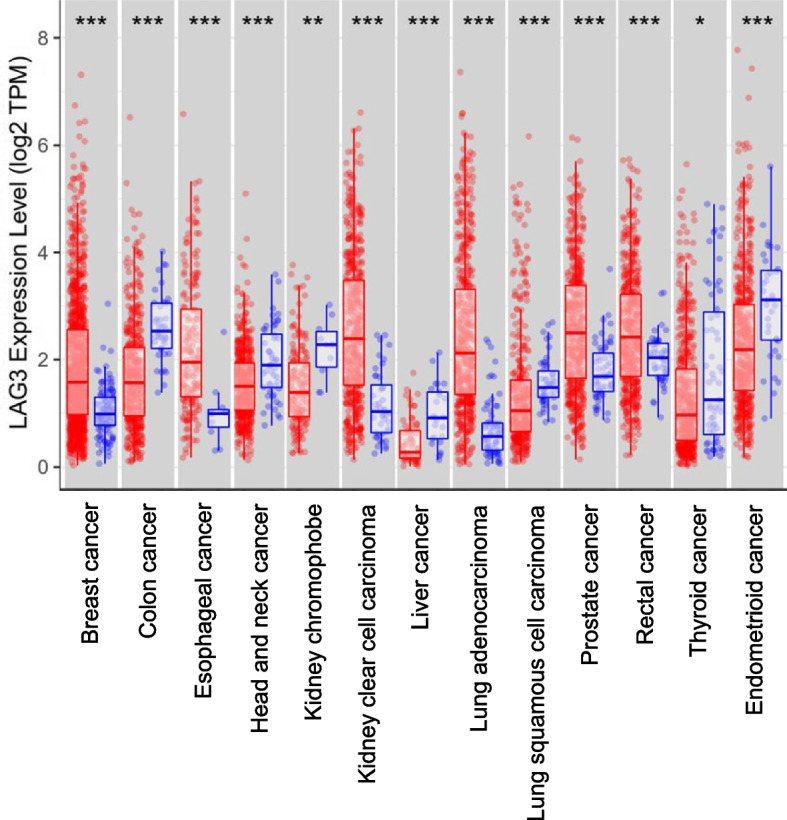
*LAG3* mRNA expression analysis in multiple cancers Comparison of *LAG3* mRNA expression between tumor and normal tissue in various human cancers with TIMER to visualize and analyze data from TCGA and analysis using the Wilcoxon rank-sum test. *LAG3* mRNA expression was higher in breast cancer, esophageal cancer, head and neck cancer, kidney clear cell carcinoma, lung adenocarcinoma, and lung squamous cell carcinoma than in normal tissues. However, *LAG3* mRNA expression was lower in colon cancer, kidney chromophobe, liver cancer, prostate cancer, rectal cancer, thyroid cancer, and endometrioid cancer than in normal tissues. Red: tumor, Blue: normal tissue, ***P < 0.001, **P < 0.01, *P < 0.05

To evaluate whether *LAG3* mRNA correlates with tumors, we compared *LAG3* mRNA expression between normal and tumor tissues from multiple cancers in TCGA. *LAG3* mRNA expression was higher in breast cancer (*P* < 0.001), esophageal cancer (*P* < 0.001), head and neck cancer (*P* < 0.001), kidney clear cell carcinoma (*P* < 0.001), lung adenocarcinoma (*P* < 0.001), and lung squamous cell carcinoma (*P* < 0.001) than in normal tissues. Conversely, *LAG3* mRNA expression was lower in the following: colon cancer (*P* < 0.001), kidney chromophobe (*P* < 0.001), liver cancer (*P* < 0.001), prostate cancer (*P* < 0.001), rectal cancer (*P* < 0.01), thyroid cancer (*P* < 0.05), and endometrioid cancer (*P* < 0.001), than in normal tissues (Fig. 1).

### Prognostic Potential of *LAG3* mRNA in Various Cancers in TCGA Cohort

As TCGA cohort revealed that *LAG3* mRNA is expressed at higher or lower levels in cancers than in normal tissues, we explored the prognostic potential of *LAG3* mRNA expression in multiple cancers from TCGA database. We discovered an association between *LAG3* expression and a poor or better prognosis in multiple cancers (Table 1). Elevated expression of *LAG3* was associated with a poor prognosis in kidney clear cell carcinoma (*P* < 0.0001), lower-grade glioma (*P* = 0.0302), ocular melanoma (*P* < 0.0001), and lower-grade glioma and glioblastoma (*P* < 0.0001). Furthermore, our results indicated that elevated expression of *LAG3* was associated with a better prognosis in melanoma (*P* < 0.0001) and thyroid cancer (*P* = 0.0149). These results suggest that *LAG3* is a novel prognostic biomarker for these cancers.

**Table 1:** *LAG3* association with prognosis of multiple cancers in TCGA database

**Cancer type**	**N**	**Biomarker**	**Poor prognosis**	***P*** **value**
Kidney clear cell carcinoma	606	mRNA	OS	< 0.0001
	604	mRNA	PFS	0.0433
Kidney papillary cell carcinoma	321	mRNA	PFS	0.0293
Lower-grade glioma	528	mRNA	OS	0.0302
Ocular melanomas	80	mRNA	OS	< 0.0001
	79	mRNA	PFS	0.0056
Pan-cancer	10952	mRNA	OS	0.0138
Lower-grade glioma and glioblastoma	694	mRNA	OS	< 0.0001
	694	mRNA	PFS	< 0.0001
**Cancer type**	**N**	**Biomarker**	**Better prognosis**	***P*** **value**
Bladder cancer	426	mRNA	PFS	0.0307
Liver cancer	422	mRNA	PFS	0.0049
Melanoma	458	mRNA	OS	< 0.0001
	459	mRNA	PFS	0.0049
Thyroid cancer	572	mRNA	OS	0.0149

*N* Number of patients, *TCGA* The Cancer Genome Atlas, *OS* Overall survival, *PFS* Progression-free survival

### Prognostic Potential of *LAG3* mRNA in MPM from TCGA Cohort

**Fig. 2:**
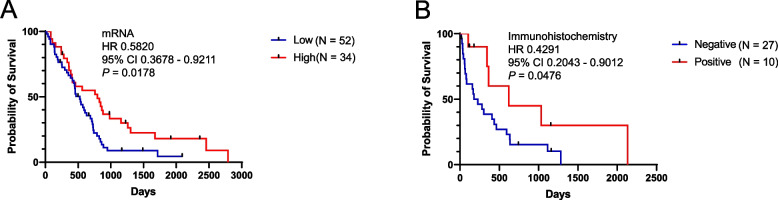
*LAG3* is associated with a better prognosis in mesothelioma. Overall survival analysis using the Kaplan–Meier method for *LAG3* mRNA (**A**) and immunohistochemical expression (**B**) groups in MPM. The log-rank test demonstrated that elevated *LAG3* mRNA and immunohistochemical expression correlated with a better prognosis (mRNA: HR = 0.5820, 95% CI 0.3678–0.9211, *P* = 0.0178, protein: HR = 0.4291, 95% CI 0.2043–0.9012, *P* = 0.0476). HR with 95% CI was reported using a univariate Cox regression model. *LAG3* mRNA expression is from TCGA, and immunohistochemical expression is from our cohort. HR, hazard ratio; CI, confidence interval; MPM, malignant pleural mesothelioma; TCGA, The Cancer Genome Atlas

As the results of TCGA cohort analysis demonstrated that *LAG3* was associated with prognosis in multiple cancers, we tested the significance of *LAG3* as a potential biomarker in MPM. We assessed *LAG3* mRNA expression in 86 patients with MPM from TCGA to evaluate the association between *LAG3* expression and clinical variables. Patient characteristics, including age, sex, stage, histology, and high/low *LAG3* mRNA expression, are summarized in Table 2. The cut-off value set as the mean for high/low LAG3 was 7.390. In total, 34/86 (29 males, 5 females) MPM cases showed high *LAG3* expression, with 4 cases in stage I, 6 in stage II, 16 in stage III, and 8 in stage IV. *LAG3* mRNA expression was not associated with clinical variable (Table 3). OS analysis using the Kaplan–Meier method indicated that elevated *LAG3* mRNA expression and epithelioid type were correlated with better OS in MPM (HR = 0.5820, 95% CI 0.3678–0.9211, *P* = 0.0178, Fig. 2A, HR = 0.5052, 95% CI 0.2772–0.9209, P = 0.0065, Supplementary Table 1). Further OS analysis with the multivariate Cox proportional hazards model revealed that elevated *LAG3* mRNA expression indicated a better tendency for OS after adjusting for age, sex, and stage (HR = 0.8779, 95% CI 0.7643–1.0050, *P* = 0.0592, Table 4).

**Table 2:** Patients’ characteristics and results of LAG3 expression

	mRNA (*N* = 86)	IHC (*N* = 37)
Characteristics	Values	Values
Age, Mean	63.1	66.3
Sex		
Male	70 (81.6%)	32 (86.5%)
Female	16 (18.4%)	5 (13.5%)
Stage		
I	10 (11.5%)	11 (29.7%)
II	16 (18.4%)	8 (21.6%)
III	44 (51.7%)	11 (29.7%)
IV	16 (18.4%)	7 (18.9%)
Histology		
Epithelioid	57 (65.5%)	26 (70.3%)
Biphasic	23 (31.0%)	5 (13.5%)
Sarcomatoid	1 (2.3%)	5 (13.5%)
Unknown	5 (5.7%)	1 (2.7%)
LAG3		
High (mRNA), Positive (IHC)	34 (39.5%)	10 (27.0%)
Low (mRNA), Negative (IHC)	52 (60.5%)	27 (73.0%)
Smoking history		
Never		9 (24.3%)
Ex + Current		28 (75.7%)
Respiratory function test		
%VC ≥ 80		12 (32.4%)
%VC < 80		19 (51.4%)
Unknown		6 (16.2%)

*IHC* Immunohistochemistry, *VC* Vital capacity

mRNA expression is from TCGA (The Cancer Genome Atlas),

and immunohistochemical expression is from our cohort

**Table 3:** The association between LAG3 expression and clinical variables

	mRNA (*N* = 86)		IHC (*N* = 37)	
Characteristics	95% CI	*P* value	95% CI	*P* value
Age	-4.204–4.421	0.9602	-13.52–-2.253	0.0074
Sex				
Male vs Female	-1.302–0.5980	0.4633	-0.5928–0.2928	0.4962
Stage				
II vs. I	-1.557–0.8468	0.5476	-0.8717–0.0308	0.0659
III vs. I	-1.424–0.8595	0.6220	-0.8338–-0.0753	0.0212
IV vs. I	-1.254–2.016	0.6347	-0.7821–0.2626	0.3075
Histology				
Epithelioid vs Biphasic + Sarcomatoid	-0.7542–0.9437	0.8248	-0.5368–0.1214	0.2083
Smoking history				
Never vs Ex + Current			-0.5036–0.2203	0.4325
Respiratory function test				
%VC ≥ 80 vs. %VC < 80			-0.1849–0.5182	0.3407

*IHC* Immunohistochemistry, *VC* Vital capacity

mRNA expression is from TCGA (The Cancer Genome Atlas),

and immunohistochemical expression is from our cohort

**Table 4:** Multivariate Cox proportion hazards model for OS showing elevated *LAG3* immunohistochemical expression as an independent prognostic biomarker in MPM

		mRNA			IHC	
Covariate	HR	95% CI	*P* value	HR	95% CI	*P* value
Age	1.0176	0.9887–1.048	0.2374	1.0099	0.9480–1.07776	0.7616
Male/Female	0.8837	0.4837–1.6143	0.6909	0.2613	0.0812–0.8409	0.0351
Stage						
II vs. I	0.5766	0.2357–1.4105	0.2277	0.8769	0.2192–3.5076	0.8527
III vs. I	0.6688	0.3061–1.4612	0.3131	4.2209	1.0627–16.7644	0.0407
IV vs. I	0.6920	0.2855–1.6772	0.4150	14.1624	2.3848–84.1033	0.0035
LAG3	0.8779	0.7643–1.0050	0.0592	0.2255	0.0610–0.8337	0.0197

mRNA expression is from TCGA (The Cancer Genome Atlas), and immunohistochemical expression is from our cohort

*OS* Overall survival, *MPM* Malignant pleural mesothelioma, *IHC* Immunohistochemistry, *HR* Hazard ratio, *CI* Confidence interval

### Prognostic Potential of LAG3 IHC for MPM 

**Fig. 3:**
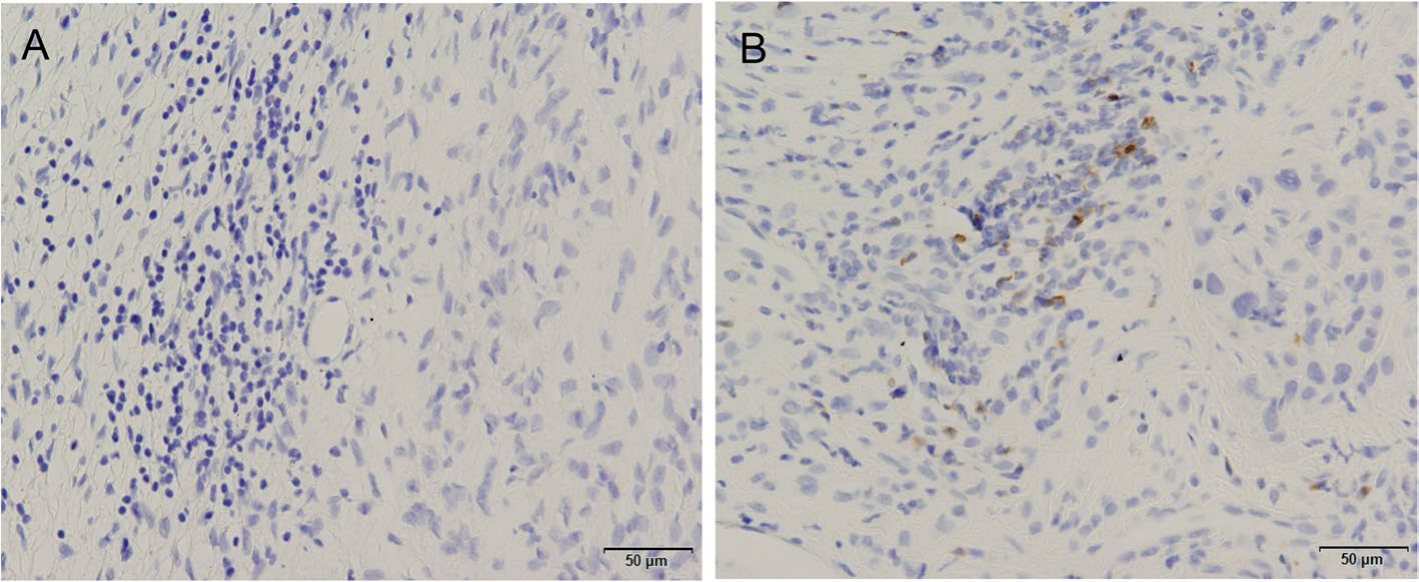
Representative immunohistochemical images of *LAG3* expression in MPM. Negative staining (**A**) and positive staining in infiltrating lymphocytes (**B**) (20 ×). *LAG3* immunohistochemical expression is from our cohort. MPM, malignant pleural mesothelioma

*LAG3* mRNA expression using TCGA indicated that elevated *LAG3* mRNA expression was correlated with better OS in MPM. Thus, we assessed immunohistochemical expression in 38 patients with MPM in our cohort to study the association between LAG3　immunohistochemical expression and clinical variables (Table 3). LAG3 expression was detected immunohistochemically in the membrane of tumor-infiltrating lymphocytes (Fig. 3) and on tumor-infiltrating CD4 or CD8 cells in MPM (Supplementary Fig. 1). However, LAG3 was not expressed in mesothelioma cells. One case was excluded from the study because the tissue was not well preserved, and the immunohistochemical results were uninterpretable. Also, 10/37 (27.0%) (8 males, 2 females) MPM cases were positive for *LAG3*: 6 cases were stage I, 1 was stage II, 1 was stage III, and 2 were stage IV. Although *LAG3* mRNA was not associated with clinical variables, LAG3 immunohistochemical expression was associated with age (95% CI -13.52–-2.253, *P* = 0.0074, Table 3) and stage I vs. III (95% CI -0.8338–-0.0753, *P* = 0.0212, Table 3). OS analysis using the Kaplan–Meier method indicated that immunohistochemical expression and stage III vs. I were correlated with better OS in MPM (HR = 0.4291, 95% CI 0.2043–0.9012, *P* = 0.0476, Fig. 2B, HR = 3.513, 95% CI 1.229–10.04, *P* = 0.0018, Supplementary Table 1).

Further OS analysis with the multivariate Cox proportional hazards model revealed that elevated LAG3 immunohistochemical expression indicated a better OS after adjusting for age, sex, and stage (HR = 0.2255, 95% CI 0.0610–0.8337, *P* = 0.0197) (Table 4). Therefore, our mRNA and immunohistochemical expression results suggest that LAG3 expression is an independent prognostic biomarker.

### Correlation Between *LAG3* mRNA Expression and TIICs in MPM in TCGA Cohort

Our study implicated LAG3 expression as an independent predictor of prognosis and a potential target for ICIs. Therefore, we assessed the correlation between *LAG3* mRNA expression and the mRNA expression of 37 gene markers of TIICs in MPM from TCGA (Table 5). *LAG3* expression was correlated with the expression of 25 of the 37 immune cell markers in MPM. Strong correlations were discovered between *LAG3* and *ICOS* (r = 0.603, *P* < 0.0001) and *CD38* expression (r = 0.6018, *P* < 0.0001). Moderate correlations were observed between *LAG3* and *CD8A* (r = 0.5744, *P* < 0.0001) and *CD8B* expression (r = 0.5594, *P* < 0.0001) and *CD80* (r = 0.5561, *P* < 0.0001). These results confirm that *LAG3* expression is correlated with various TIICs in MPM. Although OS analysis using the Kaplan–Meier method indicated that *PDCD1* and *CTLA4* expression were not correlated with OS (*PDCD1*: HR = 1.114, 95% CI 0.7043–1.763, *P* = 0.6384, *CTLA4*: HR = 1.326, 95% CI 0.8375–2.098, *P* = 0.2133) in MPM, the standard therapy for MPM is a combination ICIs with nivolumab and ipilimumab [12]. In addition, we discovered moderate correlations between *LAG3* and *PDCD1* (also known as PD-1) (r = 0.5508, *P* < 0.0001) and *CTLA4* expression (r = 0.5783, *P* < 0.0001) and a weak correlation between *LAG3* and *CD274* (also known as PD-L1) expression (*r* = 0.3971, *P* < 0.001) (Supplementary Fig. 2). Further analysis to identify factors affecting correlation of *LAG3* mRNA expression with a multivariate linear regression model revealed that *LAG3* mRNA expression was relevant to *PDCD1* (β = 0.4915, 95% CI 0.1926 – 0.7905, *P* = 0.0016) (Table 6) and *CD38* (β = 0.3021, 95% CI 0.0889 – 0.5152, *P* = 0.0061) (Table 6) after adjusting for 8 gene markers. PD-1 is an immune checkpoint having corresponding ICIs. Therefore, our results suggest that *LAG3* expression regulates the tumor immune system and could affect ICIs therapy.

**Table 5:** mRNA correlation between *LAG3* and gene markers of TIICs in MPM from TCGA

Immune cells	Gene markers	r	*P* value
Immune checkpoint	*CD274*	0.3971	0.0001
	*PDCD1*	0.5508	< 0.0001
	*CTLA4*	0.5783	< 0.0001
Macrophages	*CD68*	0.5062	< 0.0001
M1-type (classically activated) macrophages	*NOS2*	-0.0912	0.4007
M2-type (alternatively activated) macrophages	*ARG1*	0.1393	0.1982
	*MRC1*	0.0992	0.3604
Tumor-associated macrophages	*HLA-G*	0.4517	< 0.0001
	*CD80*	0.5561	< 0.0001
	*CD86*	0.4983	< 0.0001
Monocytes	*CD14*	0.303	0.0043
Natural killer cells	*XCL1*	0.4609	< 0.0001
	*KIR3DL1*	0.2702	0.0114
	*CD7*	0.3896	0.0002
Neutrophils	*MPO*	0.3458	0.001
Dendritic cells	*CD1C*	-0.1125	0.2997
B cells	*CD19*	0.1919	0.0749
	*CD38*	0.6018	< 0.0001
CD8 + T cells	*CD8A*	0.5744	< 0.0001
	*CD8B*	0.5594	< 0.0001
Follicular helper T cells	*CXCR5*	0.3631	0.0005
	*ICOS*	0.603	< 0.0001
	*BCL6*	-0.2906	0.0063
T helper-1 cells	*IL12RB2*	0.3758	0.0003
T helper-2 cells	*CCR3*	0.1533	0.1563
	*STAT6*	0.0411	0.7055
	*GATA3*	0.2013	0.0615
T helper-9 cells	*TGFBR2*	-0.1495	0.1668

**Table 6:** mRNA correlation between *LAG3* and gene markers of TIICs in MPM from TCGA with a multivariate linear regression model

Gene markers	β	95% CI	*P* value
*CD274*	0.0839	-0.0753–0.2433	0.2978
*PDCD1*	0.4915	0.1926–0.7905	0.0016
*CTLA4*	-0.1120	-0.4940–0.2700	0.5611
*CD80*	0.2795	-0.0476–0.6068	0.0930
*CD38*	0.3021	0.0889–0.5152	0.0061
*CD8A*	-0.3154	-0.8362–0.2054	0.2316
*CD8B*	0.2144	-0.2179–0.6466	0.3265
*ICOS*	0.0611	-0.3994–0.5215	0.7924

*TIICs* Tumor immune infiltration cells, *MPM* Malignant pleural mesothelioma, *TCGA* The Cancer Genome Atlas, *β* Regression coefficient

## Discussion

Accumulating evidence has indicated that LAG3 plays an important role in the immune system [13–16] and survival in various cancers [13, 17]. In this study, we revealed that *LAG3* mRNA is expressed at higher or lower levels in cancers than in normal tissues and demonstrated that *LAG3* was associated with prognosis in multiple cancers. We described *LAG3* prognostic implications for MPM and *LAG3* correlation with various gene markers of TIICs, including immune checkpoints, in MPM. Specifically, *LAG3* mRNA was expressed at a higher level in tumors than in normal tissues, and higher *LAG3* mRNA levels were associated with poor prognosis in kidney clear cell carcinoma. In contrast, *LAG3* mRNA was expressed at a lower level in tumors than in normal tissues, and higher *LAG3* mRNA levels were associated with a better prognosis in liver and thyroid cancer. The variation between the types of cancer should show a diverse role of *LAG3* for cancer.

Furthermore, we established that elevated *LAG3* mRNA and immunohistochemical expression levels were correlated with better OS in patients with MPM (Fig. 2A, B). The multivariate Cox proportional hazards model showed that LAG3 immunohistochemical expression was an independent prognostic biomarker. *LAG3* mRNA expression demonstrated a similar tendency (Table 4). One IHC study using different antibodies (#40,465, clone 11E3, 1:100, mouse monoclonal, Abcam) to estimate LAG3 immunohistochemical expression revealed no expression in MPM tissues [28]. Another study using flow cytometry to evaluate the prognostic significance of LAG3 indicated poor survival with high CD4 + LAG3 levels compared with low CD4 + LAG3 levels in MPM tissues [19]. We hypothesized that the cause of variation between our results and previous reports was related to the application of different antibodies, evaluation methods, and LAG3 targets.

Moreover, the multivariate Cox proportional hazards model confirmed that sex and stage were prognostic factors for LAG3 immunohistochemical expression. However, sex and stage were not prognostic factors for *LAG3* mRNA expression in MPM. The plausible reason for the variation between positive LAG3 IHC and high *LAG3* mRNA results should be the proportion of stage I and females in stage I. Of the 10 positive LAG3 IHC cases, 6 were stage I, and 2 patients were females, whereas 4 cases of 34 high *LAG3* mRNA cases were stage I, and none were female. These factors likely affected the results.

In addition, *LAG3* mRNA expression correlated with gene markers of TIICs in MPM, as shown in Table 5. The correlation between *LAG3* and these gene markers in TIICs indicates that *LAG3* regulates the tumor immune system in MPM. The highest correlation, i.e., a strong correlation, was observed between *LAG3* and the follicular helper T cell marker, *ICOS*; a previous study demonstrated that *ICOS* enhances the efficiency of CD8 + T cells [29]. The gene markers of CD8 + T cells, including *CD8A* and *CD8B*, major cancer cell killers [30], were also moderately correlated with *LAG3*. Accordingly, *CD8A* and *CD8B*, enhanced by *ICOS*, alter the tumor immune system, eventually contributing to a better prognosis in patients with MPM.

Moreover, *LAG3* was moderately associated with *PDCD1*, which is known as an immune checkpoint. *PDCD1* was the most relevant to *LAG3* with the multivariate linear regression model in gene markers of TIICs on MPM. Therefore, the tumor microenvironment in MPM should be considered adaptive immune resistance driven by *PD-1*/*PD-L1*, which was classified as a group of TIICs enrichment [31, 32]. The situation with the tumor microenvironment in MPM might result in multiple correlations between *LAG3* and the gene markers of TIICs. LAG3 inhibitor monotherapy resulted in fewer reductions in tumor proliferation than did PD-1 inhibitor monotherapy in an in vivo study [33]. However, the combination of LAG3 and PD-1 inhibitors drastically restricted tumor proliferation compared with LAG3 or PD-1 inhibitor monotherapy in a mouse model [33]. The combination of LAG3 and PD-1 inhibitors (relatlimab and nivolumab) is more effective than nivolumab monotherapy as a first-line therapy for advanced melanoma [13]. Although the proportion of PD-1 immunohistochemical expression was irrelevant to the results, elevated LAG3 immunohistochemical expression was associated with PFS [13]. Our results showed that high expression of *LAG3* mRNA was associated with a better prognosis in terms of OS and PFS in patients with melanoma (Table 1), consistent with previous report [17]. Our findings suggested that elevated *LAG3* mRNA and immunohistochemical expression were associated with better OS. This indicates that LAG3 immunohistochemical expression is an independent prognostic biomarker for patients with MPM. Additionally, *PDCD1* mRNA expression was the most relevant to *LAG3* mRNA expression among gene markers of TIICs in MPM. Based on our results and previous clinical and in vivo studies [13, 17, 33], LAG3 may be a potential target for ICIs in MPM, and LAG3 and PD-1 inhibitors may contribute to a better prognosis of patients with MPM.

Although we obtained multiple results for LAG3, this study had some limitations. First, this study was performed retrospectively, and the number of patients was small; therefore, prospective randomized controlled trials with large study populations are needed to evaluate the results. Second, although we integrated the data for patients with MPM relative to mRNA and immunohistochemical expression across the two databases, a study with an identical database should be performed to compare the results, including the discrepancy regarding male/female composition and stage with multivariate Cox proportion hazards model for OS between *LAG3* mRNA and LAG3 IHC. Third, this study was based on clinical research; thus, in vivo and in vitro studies should be carried out to elucidate the role and significance of LAG3 in MPM.

## Conclusions

Our results demonstrated that *LAG3* was expressed in and correlated with the prognoses of multiple cancers, especially MPM. LAG3 is an independent prognostic biomarker in patients with MPM. Our results indicate that *LAG3* is correlated with various gene markers of TIICs, including other immune checkpoints (especially PD-1). Therefore, LAG3 regulates the tumor immune system in MPM and could be a potential target for ICIs; LAG3 and PD-1 inhibitors could contribute to a better prognosis of patients with MPM. A combination of two ICIs should be tested and confirmed for obtaining better prognostic outcomes in the near future.

### Supplementary Information

**Additional file 1.**

**Additional file 2.**

**Additional file 3.**

## Data Availability

The original contributions presented in this study are included in the article/Supplementary Material. Further inquiries can be directed to the corresponding author. The data from TCGA (https://xena.ucsc.edu/) and our cohort were utilized during the study can be obtained from the corresponding author upon request.

## Abbreviations

MPM: Malignant pleural mesothelioma
TCGA: The Cancer Genome Atlas
OS: Overall survival
PFS: Progression-free survival
