# Supplementary material for: LAG3 is an independent prognostic biomarker and potential target for immune checkpoint inhibitors in malignant pleural mesothelioma: a retrospective study
Source: BMC Cancer. 2023 Dec 7;23:1206. doi: 10.1186/s12885-023-11636-1 (PMC10704683; doi:10.1186/s12885-023-11636-1)
Supplement: Supplementary file 1 — Additional file 1. [file 12885_2023_11636_MOESM1_ESM.pptx]

## Slide 1
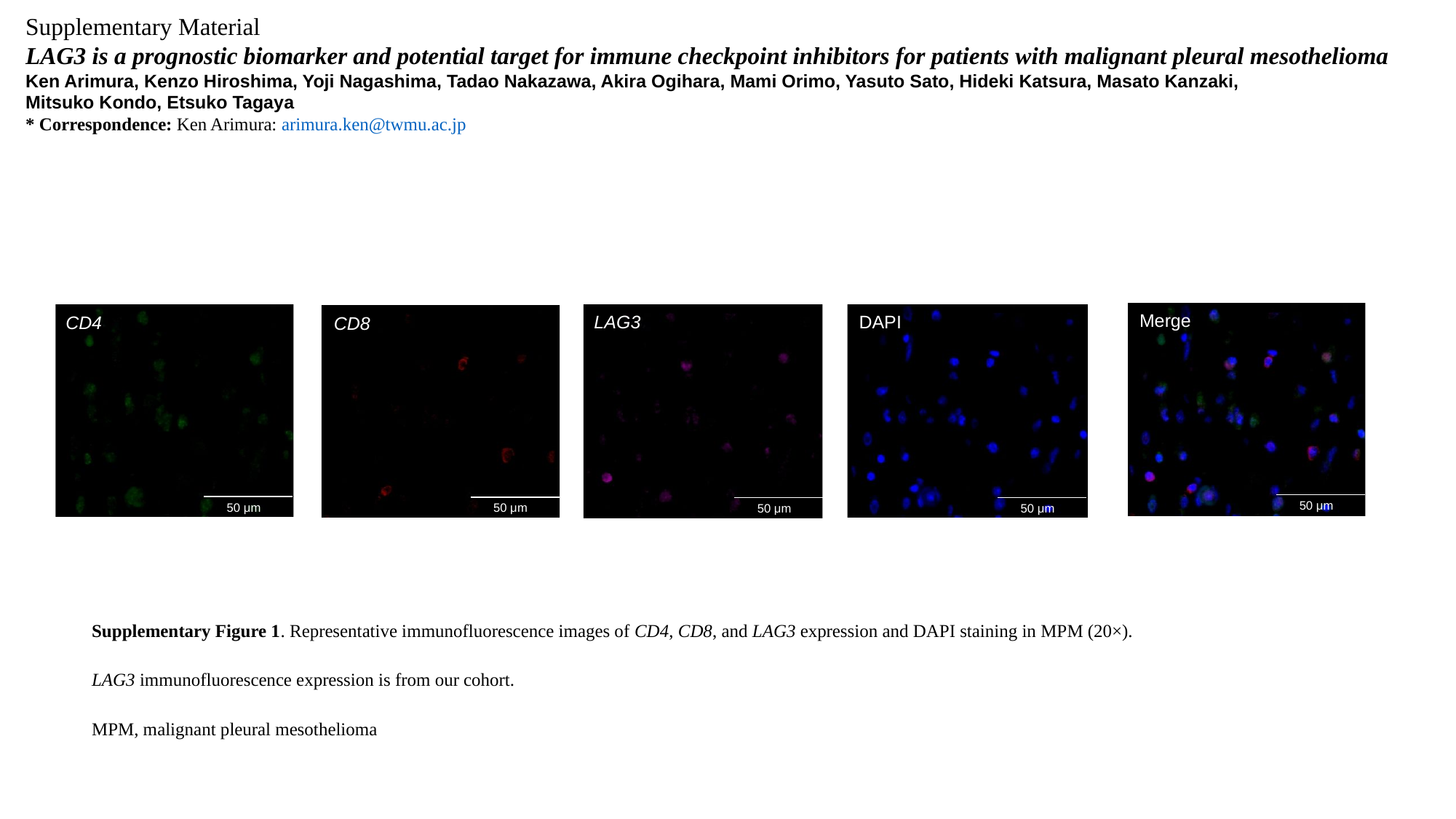

Supplementary Material
LAG3 is a prognostic biomarker and potential target for immune checkpoint inhibitors for patients with malignant pleural mesothelioma
Ken Arimura, Kenzo Hiroshima, Yoji Nagashima, Tadao Nakazawa, Akira Ogihara, Mami Orimo, Yasuto Sato, Hideki Katsura, Masato Kanzaki,
Mitsuko Kondo, Etsuko Tagaya
* Correspondence: Ken Arimura: arimura.ken@twmu.ac.jp
Merge
LAG3
DAPI
CD4
CD8
50 μm
50 μm
50 μm
50 μm
50 μm
Supplementary Figure 1. Representative immunofluorescence images of CD4, CD8, and LAG3 expression and DAPI staining in MPM (20×).
LAG3 immunofluorescence expression is from our cohort.
MPM, malignant pleural mesothelioma
