# Supplementary material for: LAG3 is an independent prognostic biomarker and potential target for immune checkpoint inhibitors in malignant pleural mesothelioma: a retrospective study
Source: BMC Cancer. 2023 Dec 7;23:1206. doi: 10.1186/s12885-023-11636-1 (PMC10704683; doi:10.1186/s12885-023-11636-1)
Supplement: Supplementary file 2 — Additional file 2. [file 12885_2023_11636_MOESM2_ESM.pptx]

## Slide 1
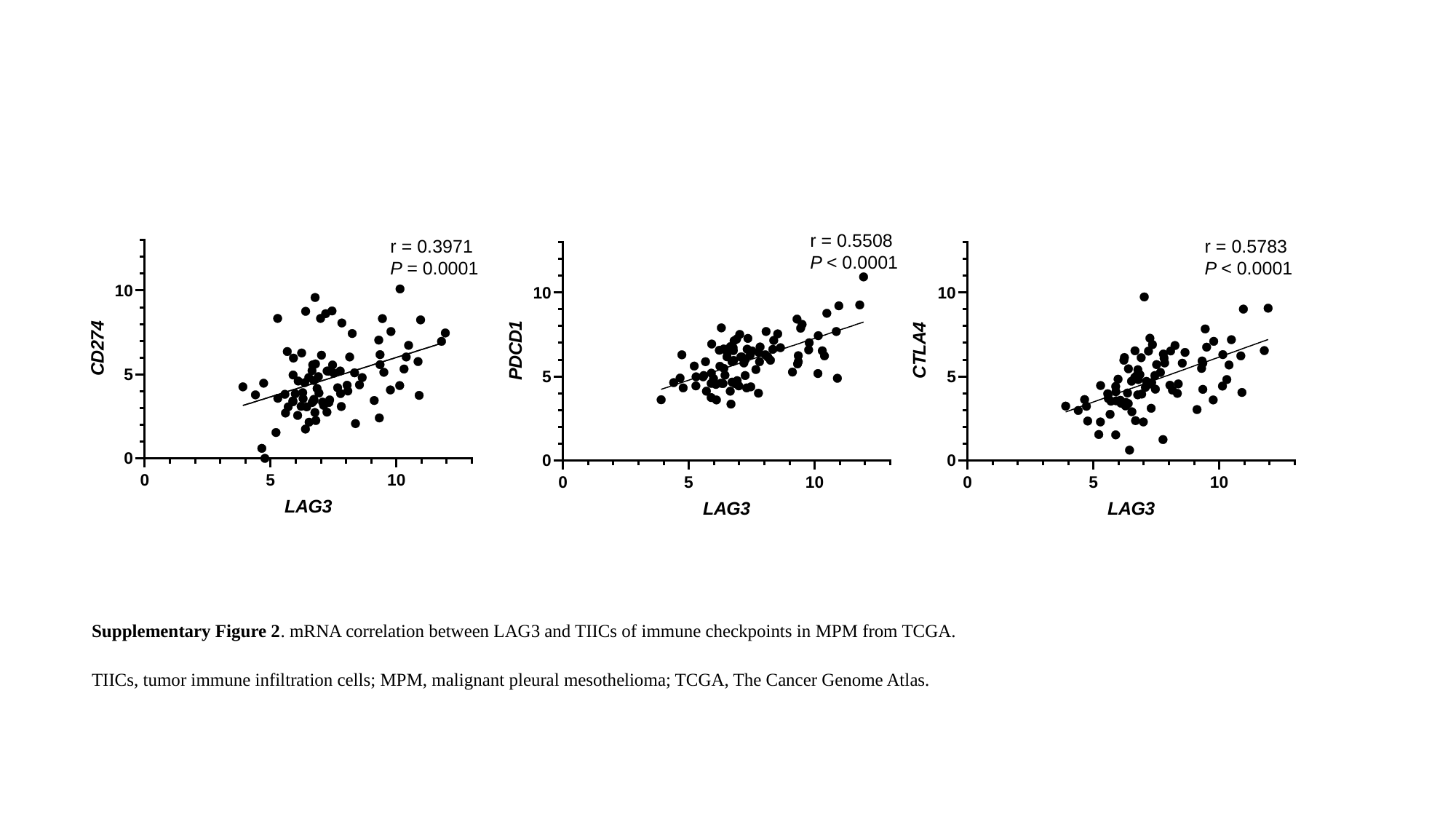

r = 0.5508
P < 0.0001
r = 0.5783
P < 0.0001
r = 0.3971
P = 0.0001
Supplementary Figure 2. mRNA correlation between LAG3 and TIICs of immune checkpoints in MPM from TCGA.
TIICs, tumor immune infiltration cells; MPM, malignant pleural mesothelioma; TCGA, The Cancer Genome Atlas.
