# Supplementary material for: LAG3 is an independent prognostic biomarker and potential target for immune checkpoint inhibitors in malignant pleural mesothelioma: a retrospective study
Source: BMC Cancer. 2023 Dec 7;23:1206. doi: 10.1186/s12885-023-11636-1 (PMC10704683; doi:10.1186/s12885-023-11636-1)
Supplement: Supplementary file 3 — Additional file 3. [file 12885_2023_11636_MOESM3_ESM.pptx]

## Slide 1
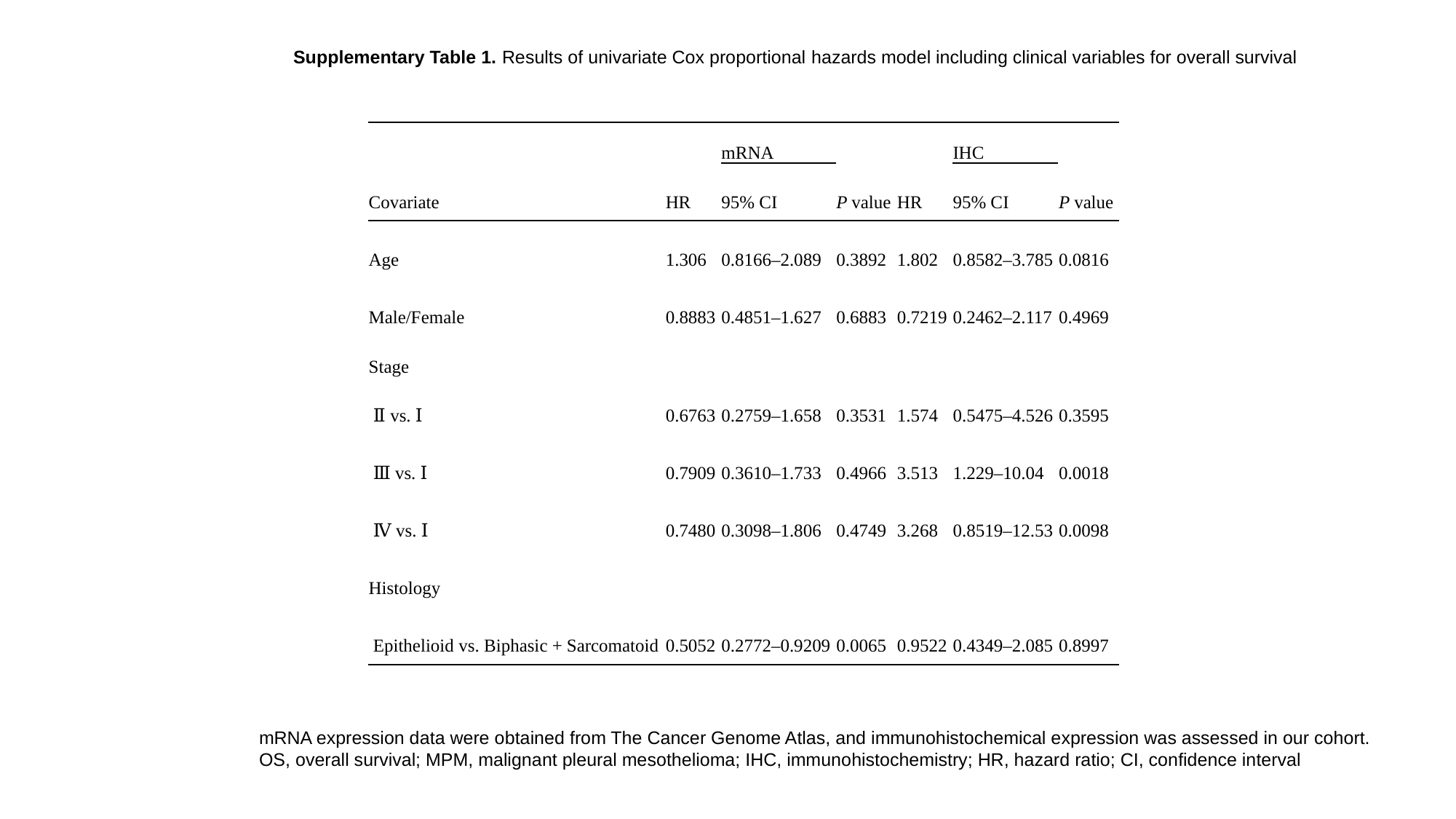

Supplementary Table 1. Results of univariate Cox proportional hazards model including clinical variables for overall survival
| | | | | | | |
| --- | --- | --- | --- | --- | --- | --- |
| | | mRNA | | | IHC | |
| Covariate | HR | 95% CI | P value | HR | 95% CI | P value |
| Age | 1.306 | 0.8166–2.089 | 0.3892 | 1.802 | 0.8582–3.785 | 0.0816 |
| Male/Female | 0.8883 | 0.4851–1.627 | 0.6883 | 0.7219 | 0.2462–2.117 | 0.4969 |
| Stage | | | | | | |
| Ⅱ vs. Ⅰ | 0.6763 | 0.2759–1.658 | 0.3531 | 1.574 | 0.5475–4.526 | 0.3595 |
| Ⅲ vs. Ⅰ | 0.7909 | 0.3610–1.733 | 0.4966 | 3.513 | 1.229–10.04 | 0.0018 |
| Ⅳ vs. Ⅰ | 0.7480 | 0.3098–1.806 | 0.4749 | 3.268 | 0.8519–12.53 | 0.0098 |
| Histology | | | | | | |
| Epithelioid vs. Biphasic + Sarcomatoid | 0.5052 | 0.2772–0.9209 | 0.0065 | 0.9522 | 0.4349–2.085 | 0.8997 |
mRNA expression data were obtained from The Cancer Genome Atlas, and immunohistochemical expression was assessed in our cohort.
OS, overall survival; MPM, malignant pleural mesothelioma; IHC, immunohistochemistry; HR, hazard ratio; CI, confidence interval
